# Phenotypic and Molecular Characterization of Bovine Mastitis Milk Origin Bacteria and Linkage of Intramammary Infection With Milk Quality

**DOI:** 10.3389/fvets.2022.885134

**Published:** 2022-06-02

**Authors:** Zul I. Huma, Neelesh Sharma, Savleen Kour, Sung Jin Lee

**Affiliations:** ^1^Division of Veterinary Medicine, Faculty of Veterinary Sciences and Animal Husbandry, Sher-e-Kashmir University of Agricultural Sciences & Technology of Jammu, Jammu, India; ^2^Department of Animal Biotechnology, College of Animal Life Sciences, Kangwon National University, Chuncheon-si, South Korea

**Keywords:** bacterial isolates, milk quality, somatic cell count, toxic genes, mastitis

## Abstract

Mastitis is a multi-etiological complex disease of dairy cows and negatively affects the quality and quantity of milk. Milk is a nutritious food for human being; milk quality is negatively affected by intramammary infection of dairy cows. A total of 300 milk samples were collected from mastitis dairy cows irrespective of parity and stage of lactation, 235 (78.33%) samples were culturally positive and yielded 1,100 bacterial isolates. *Staphylococcus aureus* was found to be the prime etiological agent involved in the mastitis of dairy cows, followed by *Escherichia coli* and other environmental pathogens. On the molecular characterization of isolates obtained from the milk culture, various toxic genes such as *nuc, seb, hla, stx*1, *stx*2, *hly*, and *Sagl* were found on different isolated bacteria. Milk somatic cell counts (SCC) were found to be directly related to the severity of mastitis. On drawing the SCC correlation with milk components, it was found that SCC had a significant negative correlation with fat, lactose, solid not fat (SNF), and ash. It was concluded that mastitis-affected milk contains numerous pathogenic bacteria, toxins, and reduced milk quality, which is unfit for human consumption.

## Introduction

Mastitis is an inflammation of the mammary parenchyma, characterized by physical and chemical changes in the gland tissue and glandular secretions, respectively. It is most commonly a result of an invasion of the pathogen in the mammary gland (1). It is a cause of major economic burden for the dairy industry in the form of loss of milk, quarter, and treatment costs. A diverse group of organisms is involved in the mammary gland inflammation, and variations in the prevalence may be found between countries. The majority of mastitis is of bacterial origin worldwide, however, only few species are responsible for most of the cases, such as *Staphylococcus aureus, Escherichia coli, Streptococcus uberis, Streptococcus dysgalactiae, Streptococcus agalactiae* (2).

Organisms classified as environmental bacteria can survive in the mammary gland and thus are capable of causing subclinical infections, manifested as an increase in milk somatic cell count (SCC) (3). The SCC of the milk is a count of cells, including the epithelial and inflammatory ones, thus acts as an indicator and predictor of intramammary inflammation/ mastitis (4). An elevated SCC in raw milk has a negative correlation with its quality. Major mastitis-causing bacteria, including *S. aureus* and *E. coli* have the ability to produce toxins that serve as an important cause of food poisoning. Food poisoning caused by *S. aureus* is related to the production of staphylococcal enterotoxins (SE), which act on-specific, emetic receptors located in the intestinal wall. Enterotoxins are short, water-soluble, and extracellular proteins. More importantly, many of these toxins have been found resistant to higher temperatures, such as those employed during the pasteurization process (5). Toxin genes and virulence factors play a significant role in pathogenesis and antibiotic resistance to various antimicrobial agents (6). The association between different virulence factors, clinical manifestations, and milk quality impacts is still barely known.

Elucidating the distribution pattern of mastitis-causing microorganisms in the milk and their virulence factors can support the advancement of strategized control of mastitis and help reduce the possibilities of food poisoning. There is no such detailed data available in Jammu region of UT Jammu &Kashmir, India on genotypic characterization and toxin-producing genes of mastitis-causing bacteria's with its impact on milk quality.

Pathogens inside the mammary gland initiate the inflammatory reaction, damage epithelial cells, and increase blood capillaries' permeability. As a result, although not always predictable, milk composition changes depending on various factors such as severity and duration of infection, disruption of epithelial cell integrity, and other secondary physiological changes (7).

Therefore, this study was planned to isolate and genotypically characterize mastitis milk origin microorganisms and assess the presence of genes related to toxin production. Apart from this, the study also focuses on understanding the impact of intramammary infection on milk quality by assessing milk SCC and milk composition.

## Methods

### Animals and Sample Collection

Cows maintained on the organized and unorganized cattle farms were milked twice a day manually in the morning and evening in the presence of their calves. Unorganized farms herein are designated usually for individual farmers who rear 1–4 animals (cows) only. Hygienic practices observed at the farms were very poor, and only a few farmers adopted the practice of regular washing and disinfection of the teats/udder prior to milking, and even clean clothes were not used for drying off the udder. Moreover, farmers were unaware of mastitis cow side tests such as California Mastitis Test (CMT). The farmers rarely followed the practice of teat dips (pre- or post-dipping) for the prevention and control of mastitis.

A cow side mastitis that is CMT was performed to test the presence of infection in the milk at the farms. A total of 300 cows from various farms were screened for the CMT test and the composite milk samples from all the quarters of cow were taken who gave score of ≥2 in CMT for mastitis in Jammu region of UT Jammu & Kashmir, India. The samples were collected irrespective of the cow's parity and stage of lactation and were transported to the lab in ice for further processes. In the laboratory, the samples were subjected to determine somatic cell count, milk constituents, and the impact of mastitis-causing bacteria on the quality of milk after gentle mixing. The milk samples were then processed for culture, isolation, and identification of their isolates.

### Somatic Cell Count

Milk samples were analyzed for milk SCC in the laboratory using Automatic milk somatic cell counter DCC (Milkoscan, DeLaval, Sweden). Samples were analyzed as per the given instructions supplied in the manual within 2 h of collection. SCC results were expressed in × 10^3^ /mL of sample.

### Milk Composition

Milk samples collected were subjected to estimate different milk constituents such as fat, lactose, protein, ash, and solid not fat (SNF) by using Lactoscan Milk Analyser (Nune's, India).

### Isolation and Identification of Bacteria

Milk samples were subjected to isolation and identification of microorganisms, including bacteria, based on their morphological, cultural, and biochemical characteristics as per the National Mastitis Council procedure and Buchnan and Gibbons (8). Staining was done using Gram's Stain Kit (HiMedia, India; Cat. No. K001). In the first step, milk samples were inoculated for enrichment on Nutrient broth (HiMedia, India; Cat. No. M002) for 24 h A loopful (5 mm) of each sample from nutrient the broth was streaked on nutrient agar (HiMedia, India; Cat. No. M002) and incubated the plates at 37°C for 24–36 h aerobically to record the cultural characteristic of the bacterial isolates with respect to size and shape of individual colony. An individual colony was picked up and subjected Gram's staining to identify the type of organisms. Thereafter, colonies were streaked on sheep blood agar (HiMedia, India; Cat. No. MP1301) to record the hemolysis pattern (∞/β type). On the basis of staining and hemolysis characteristics, bacterial isolates were grown on the selective media (HiMedia, India).

The pure cultures of isolated microorganisms from selective media were also subjected to biochemical characterization using the catalase test, oxidase test, coagulase test, and the growth on mannitol salt agar (MSA) (HiMedia, India; Cat. No. M118). Similarly, Gram-negative bacteria were also selectively grown on MacConkey agar (Cat. No. M081B, HiMedia, India) and EMB agar (HiMedia, India; Cat. No. M317) before subjecting them to catalase test, oxidase test, and IMViC test for further identification by using appropriate HiIMViC Biochemical Test kit (HiMedia, India; Cat. No. KB001).

### DNA Extraction and Molecular Characterization

#### DNA Extraction

Bacterial isolates were cultured in brain heart infusion (BHI) (HiMedia, India; Cat. No. M210) broth at 37°C for 24 h, and 1 mL of culture containing 10^5^ CFU/mL was transferred to sterile 2 mL tubes and centrifuged at 5,000 rpm to sediment the bacterial pellet. After removing the supernatant, the bacterial pellet was resuspended in 200 μL of nuclease free water (NFW) in 2 mL microcentrifuge tubes. The samples were boiled for 10 min, cooled on ice for 10 min, and centrifuged at 13,000 *g* in a G-star plus centrifuge (Genetix, Biotech Asia) for 5 min. Two microliters of the supernatant were used as the template for polymerase chain reaction (PCR). The concentration of DNA templates was determined spectrophotometrically with an optical density at 260 nm and 280 nm. The ratio of optical density at 260/280 nm was considered to check the purity of the DNA template. Concentration of the template >1.5 ug/mL and ratio >1.5 were considered for PCR reactions.

### Genotypic Characterization

The presumptive bacterial isolates were confirmed by targeting their species-specific genes. PCR was used to amplify the *nuc* (270 bp), *E coli* (232 bp), and *S agl* (405 bp) genesto confirm *Staphylococcus aureus, E. coli*, and *Streptococcus agalactiae* using their respective primers (Table 1). Further, *S. aureus* and *E. coli* were subjected to genotypic characterization of their toxin-producing genes such as *seb, hla, stx1, stx2*, and *hly* using their specific primers (Table 1).

**Table 1:** Nucleotide sequences of the bacteria-specific or their toxic genes for PCR amplification.

**S.** **No**.	**Bacteria name**	**Gene name**	**Nucleotide sequence** **(5'– 3')**	**Size ** **(bp)**	**References**
1	*Staphylococcus aureus*	*nuc*	GCGATTGATGGTGATACGGTT AGCCAAGCCTTGACGAACTAAAGC	270	(9)
2	*Staphylococcus aureus*	*seb*	ACATGTAATTTTGATATTCGCACTG TGCAGGCATCATGTCATACCA	643	(10)
3	*Staphylococcus aureus*	*hla*	GGTTTAGCCTGGCCTTC CATCACGAACTCGTTCG	535	(11)
4	*E. coli*	*E coli*	ATCAACCGAGATTCCCCCAGT TCACTATCGGTCAGTCAGGAG	232	(12)
5	*E. coli*	*Stx1*	ATAAATCGCCATTCGTTGACTAC AGAACGCCCACTGAGATCATC	180	(13)
6	*E. coli*	*Stx2*	GGCACTGTCTGAAACTGCTCC TCGCCAGTTATCTGACATTCTG	255	(13)
7	*E. coli*	*hly*	GCATCATCAAGCGTACGTTCC AATGAGCCAAGCTGGTTAAGCT	534	(13)
8	*Streptococcus agalactiae*	*S agl*	CGCTGAGGTTTGGTGTTTACA CACTCCTACCAACGTTCT TC	405	(14)

Further on, the toxins produced by some bacteria were also characterized genotypically using their specific genes (Table 1). The PCR was performed in a 20 μL reaction using AccuPower® PCR PreMix (10 μL), which already had DNA polymerase, dNTPs, Buffer, and MgCl_2_ added. Further on 0.5 μL (10 pmol) of each forward and reverse primer, 2 μL DNA template, and 7 μL of nuclease-free water (NFW) were added.

After that, the PCR products were subjected to gel electrophoresis using a 1% (w/v) agarose gel stained with 0.5 μg/mL ethidium bromide for 45 min at a voltage of 1–5 V/cm. A 100 bp ladder was used to determine the size of the product amplified. The gel was removed and visualized until ultraviolet illumination and photographed with a gel documentation system (Mini Lumi gel documentation system, Peqlab, Germany).

### Statistical Analysis

Statistical analysis was carried out by using SPSS version 20. Descriptive statistics are presented in frequency and percentage. ANOVA test and correlation coefficient were used for the analysis of SCC and milk composition. Statistical analysis was considered significant at *p* < 0.05 or *p* < 0.01.

## Results

### Relationship of Milk SCC With Mastitis and Milk Composition

In the present study, we graded the CMT with the SCC score (Table 2). The milk samples which had SCC ≤ 200 thousand per mL of milk were thought of as negative for mastitis and CMT score was also graded as “0” (negative). Grading of CMT was done based on the severity of mastitis.

**Table 2:** Grading of milk-based on the CMT and SCC.

**S. No**.	**Mastitis status by CMT**	**CMT grading**	**Milk SCC (x10^**3**^/ml)**
1.	Negative	0	≤ 200
2.	+	1	200–800
3.	++	2	800–1,200
4.	+++	3	≥1,200

A total of 235 milk samples were subjected for the analysis of milk SCC and constituents to study the impact of mastitis on milk composition in relation to the severity of mastitis such as CMT negative, +, + +, and + + + scores. The overall results of milk composition are presented in Table 3. The present study revealed that with the increasing severity of mastitis that is CMT grading from “0” to “3,” the milk SCC was also increased significantly (*p* < 0.05) from 96.00 ± 3.60 (× 10^3^/mL) to 1738.70 ± 79.40 (× 10^3^/mL) (Table 3). Elevated SCC has also been associated with a significant (*p* < 0.05) decrease in the percentages of fat, lactose, protein, ash, and SNF in milk (Table 4).

**Table 3:** Cow milk somatic cell count (× 10^3^/mL) and other milk constituents in relation to CMT grading on the basis of severity of mastitis (mean ± SE).

**CMT grading**	**SCC (x10^**3**^/ml)**	**Fat** **(%)**	**Lactose (%)**	**Protein (%)**	**Ash (%)**	**SNF**
0	96.00 ± 3.60^a^	3.62 ± 0.00^a^	4.83 ± 0.02^a^	3.62 ± 0.02^a^	0.69 ± 0.00^a^	8.99 ± 0.03^a^
1	521.09 ± 11.26^b^	3.50 ± 0.01^b^	4.80 ± 0.01^b^	3.60 ± 0.01^a^	0.65, ± 0.01^b^	9.11 ± 0.02^b^
2	1001.80 ± 8.77^c^	3.41 ± 0.01^c^	4.58 ± 0.01^c^	3.57 ± 0.01^ab^	0.64 ± 0.01^b^	8.90 ± 0.02^c^
3	1738.70 ± 79.40^d^	3.16 ± 0.02^d^	4.43 ± 0.01^c^	3.57 ± 0.01^b^	0.62 ± 0.01^c^	8.74 ± 0.02^d^

*a,b,c,d Means with different superscripts within a row differ significantly at 5% (p < 0.05) level*.

**Table 4:** Correlations between milk SCC and milk components in lactating dairy cows.

	**Fat**	**Lactose**	**Protein**	**Ash**	**SNF**
Lactose	0.414**				
Protein	0.071*	0.087*			
Ash	0.219**	0.207**	−0.027^NS^		
SNF	0.240**	0.598**	0.551**	0.097**	
CMT	−0.502**	−0.488**	−0.055^NS^	−0.234**	−0.260**

** (P < 0.01), *(P < 0.05), NS, Non-significant.

Results showed a significant (*p* < 0.01) negative correlation between SCC and fat content (−0.502), lactose content (−0.488), ash content (−0.234), and SNF content (−0.260) (Table 4). However, the correlation between SCC and protein content (−0.055) was non-significant and negative (Table 4).

### Distribution Pattern of Isolates Obtained by Cultural Examination

**Figure 1:**
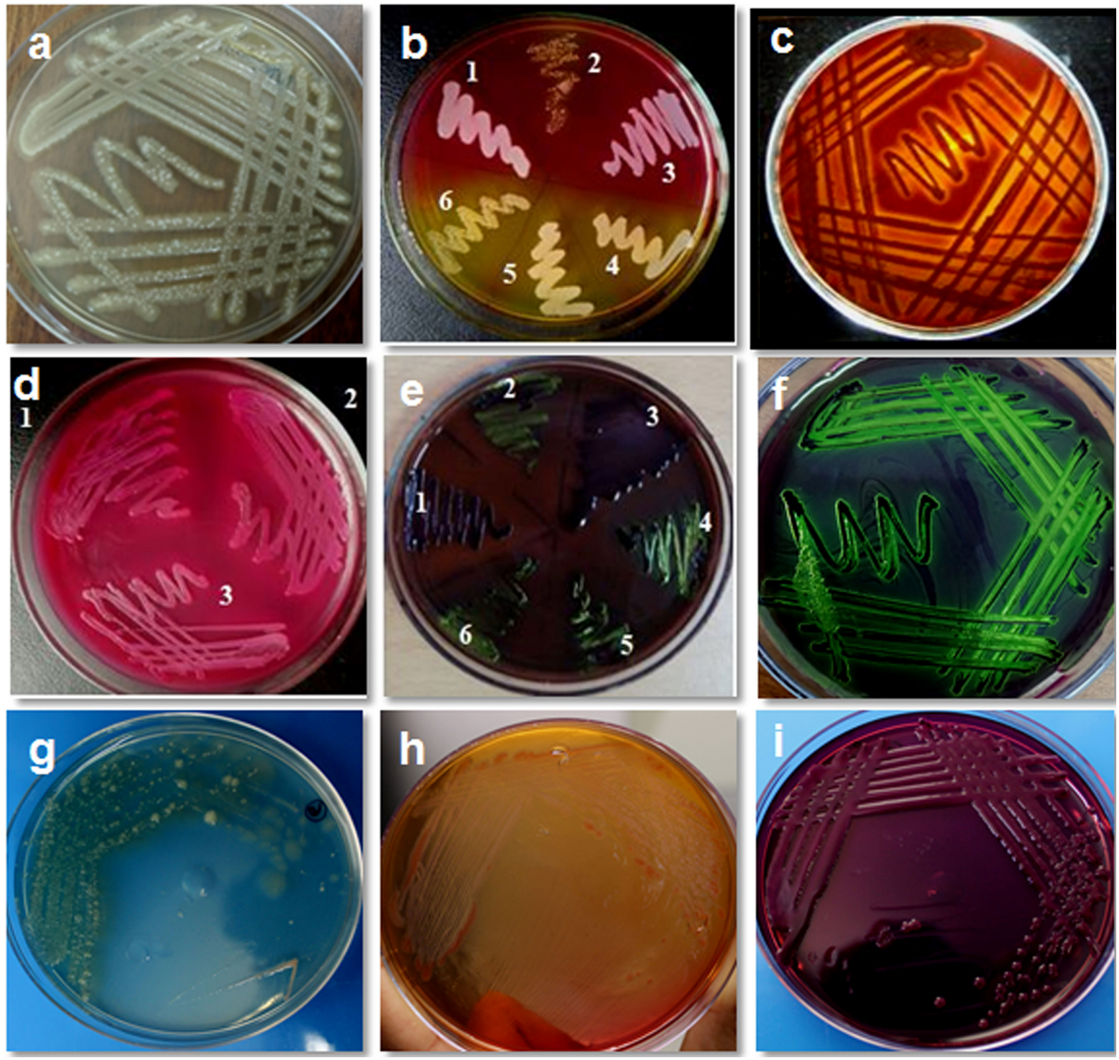
Isolation and cultural characteristics of bacterial isolates from bovine mastitis milk. **(a)** *Staphylococcus aureus* on nutrient agar showing mucoid colonies; **(b)** growth of *S. aureus* on mannitol salt agar showing yellow colonies (4,5,6) whereas *S. epidermidis* show pink colonies (1,2,3) **(c)** *S. aureus* on blood agar showing βhemolysis; **(d)** growth of *E. coli* on MacConkey agar (lactose fermenting, pink colonies); **(e)** growth of *E. coli* on EMB agar (typical green metallic sheen) sample no. 2, 4, 5, 6; **(f)** *E. coli* showing typical green metallic sheen on EMB agar; **(g)** *Pseudomonas* on nutrient agar showing blue-green colony; **(h)** *Pseudomonas* on MacConkey agar showing mucoid colony; **(i)** *Klebsiella* on EMB agar showing mucoid colony.

**Figure 2:**
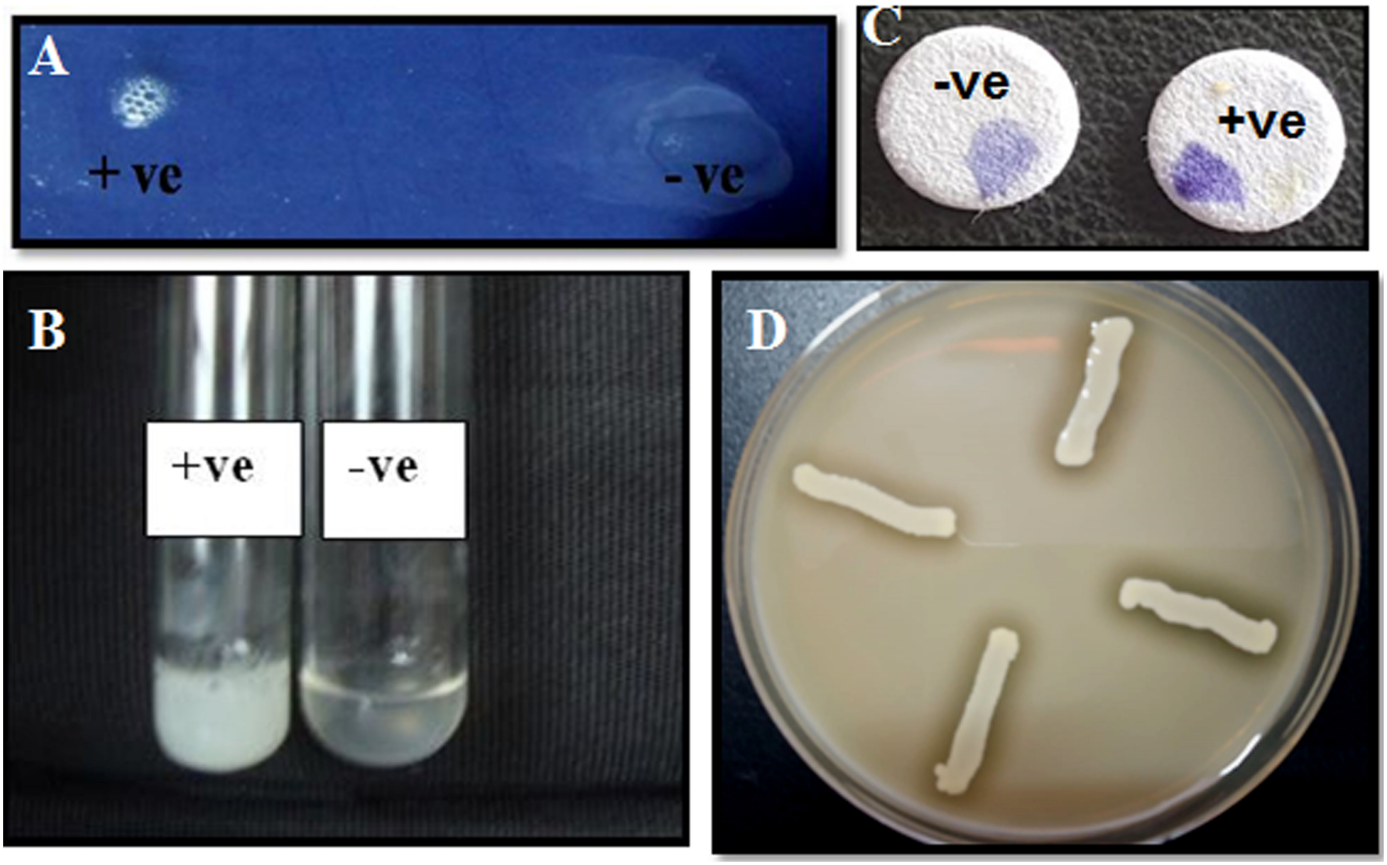
Characterization of bacterial isolates by biochemical tests. **(a)** Catalase test: Effervescence seen on the addition of *S. aureus* to 3% H_2_O_2_; **(b)** tube coagulase test: coagulation observed for *S. aureus*; **(c)** oxidase test—development of purple color on disc within 10 s of adding the organism; **(d)** halo zone observed for *S. aureus* on DNase agar.

A total of 300 cows were examined during the study; out of the milk samples collected, only 235 (78.33%) samples showed bacterial growth. On cultural examination, a total of 1,100 isolates (379 isolates of *S. aureus*, 235 isolates of *Escherichia coli*, 149 isolates of coagulase negative staphylococci, 115 isolates of *Streptococcus agalactiae*, 93 isolates of *Salmonella typhimurium*, 78 isolates of *Klebsiella pneumonia*, 18 isolates of *Enterococcus faecalis*, 15 isolates of *Proteus vulgaris*, 11 isolates of *Corynebacterium diptheriae*, and 7 isolates of *Bacillus cereus*) were obtained from 235 samples. Culture of organisms on different selective media revealed a green colony on HiCrome Staph selective depicting *S. aureus*, bluish-green colony exhibited by *E. coli* on HiCrome *E. coli* selective media, light brown colonies on HiCrome Salmonella agar revealed *Proteus vulgaris*. In contrast, light pink colonies depicted *Salmonella typhimurium* on same agar, for *Klebsiella pneumonia* purple magenta mucoid type colonies, were formed on HiCrome *Klebsiella* selective. This was also confirmed by using different media's for the selective growth of the organism like blood agar, Mannitol salt agar (MSA), MacConkey agar, and EMB agar. *S. aureus* showed round mucoid colonies on nutrient agar (Figure 1a), whereas on MSA showed yellow colonies (Figure 1b) and β hemolysis on blood agar (Figure 1c). *S. aureus* was confirmed by different biochemical examinations to be catalase (Figure 2a) and coagulase (Figure 2b) positive, whereas it was found to be oxidase negative (Figure 2c) and further confirmed by the formation of halo zone on DNase agar (Figure 2d). Similarly for *E. coli* typical green metallic sheen was observed on EMB agar (Figure 1f). Other bacteria on their selective media showed their typical characteristics, as shown in Figure 1. Confirmation of Enterobacteriaceae members was done using the IMViC test (Table 5). Among the various microorganisms isolated *Staphylococcus aureus* was the prime causative agent (36.09%, 379 isolates), followed by *Escherichia coli* (21.36%, 235 isolates), coagulase negative Staphylococci (13.54%, 149 isolates), *Streptococcus agalactiae* (10.45%, 115 isolates), *Salmonella typhimurium* (8.45%, 93 isolates), *Klebsiella pneumonia* (7.09%, 78 isolates), *Enterococcus faecalis* (1.63%, 18 isolates), *Proteus vulgaris* (1.36%, 15 isolates), *Corynebacterium diptheriae* (1.00%, 11 isolates) whereas *Bacillus cereus* (0.63%, 7 isolates) was the least prevalent amongst all.

**Table 5:** Biochemical characteristics of *Enterobacteriaceae*.

**Organism**	**Indole**	**Methyl Red**	**Vogesproskauer**	**Citrate**	**Catalase**	**Oxidase**
*E. coli*	**+**	**+**	**-**	**-**	**+**	**-**
*Enterobacteraerogenes*	**-**	**-**	**+**	**+**	**+**	**-**
*Klebsiella pneumoniae*	**-**	**-**	**+**	**+**	**+**	**-**
*Salmonella Typhi*	**-**	**+**	**-**	**-**	**+**	**-**
*Shigella*	**-**	**+**	**-**	**-**	**+**	**-**
*Proteus vulgaris*	**+**	**+**	**-**	**-**	**+**	**-**

### Distribution of Isolates Based on Staining Procedure

**Figure 3:**
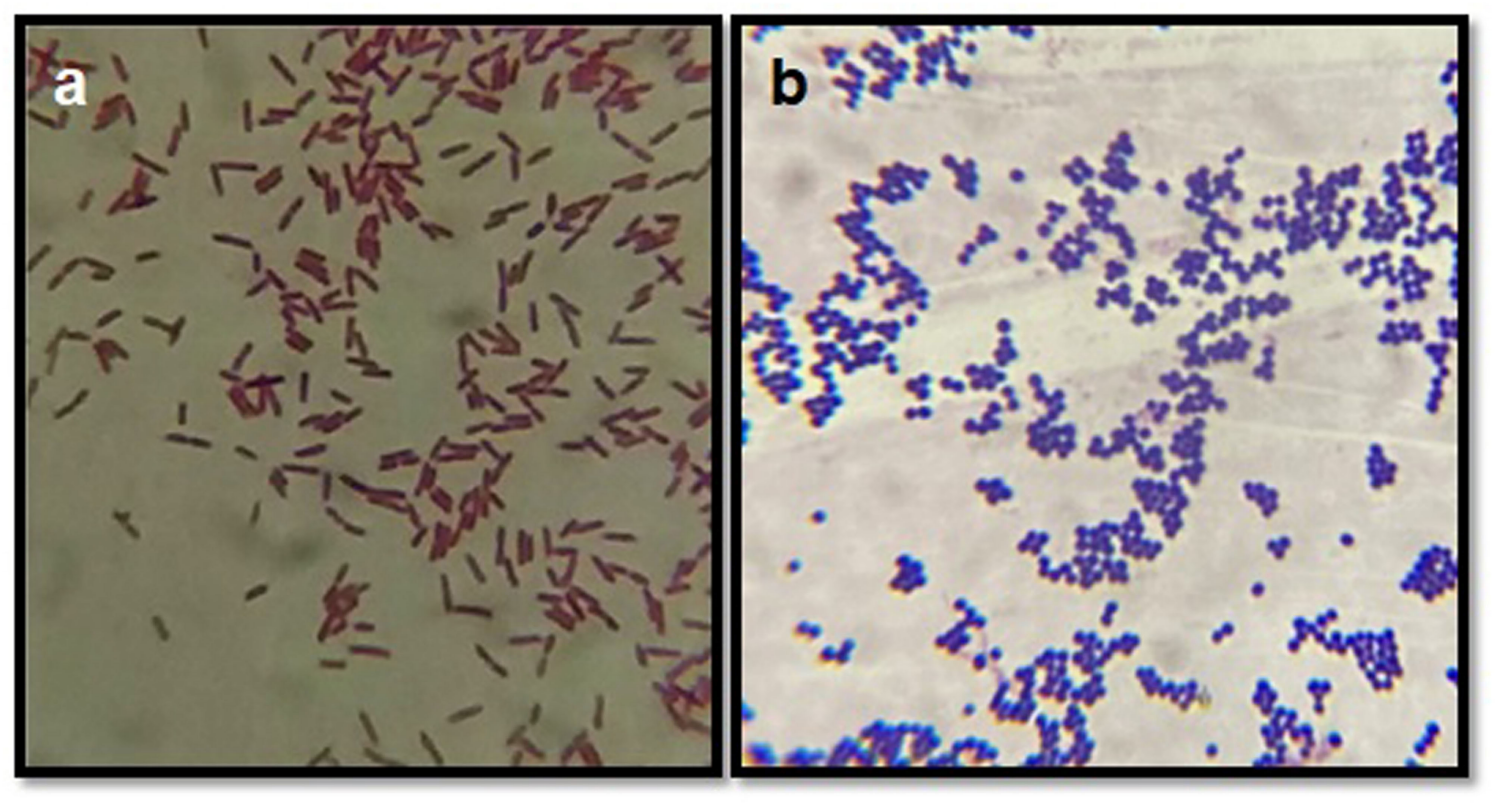
Gram's staining of bacterial isolates. **(a)** Depicts the Gram negative rods (*E. coli*) and **(b)** depicts the Gram positive cocci (bunches) *S. aureus*.

After culturing on blood agar/nutrient agar, isolates were subjected to Gram's staining, on the basis of which Gram-positive and Gram-negative organisms were differentiated (Figure 3). Based on the Gram's staining, Gram positive bacteria (*S. aureus*, CNS, *Strep. agalactiae, C. diptheriae* and *B. cereus*) were listed at the prime spot with 661 (60.09%) isolates out of the total 1,100 isolates, followed by Gram negative 439 (39.91%) isolates (*E. coli, S. typhimurium, K. pneumonia, E. faecalis, and P. vulgaris*).

### Molecular Characterization of Bacterial Isolates

On cultural examination, it was found that *S. aureus, Strep. Agalactiae* and *E. coli* were the most prevalent and commonly isolated bacteria from mastitis-affected cows. Hence they were further subjected for molecular characterization and confirmation by the PCR. Organisms like *S. aureus, Strep. Agalactiae*, and *E. coli* after cultural examination and biochemical confirmation, were further confirmed by using their specific genes such as *nuc, Sagl*, and *E. coli* genes, respectively, (Table 6). Their pathogenicity was studied by using specific toxic genes by PCR.

**Table 6:** List of bacterial and their toxin genes found positive by molecular characterization.

**Organism**	**Gene**	**Size** **(bp)**	**Total no. of isolates**	**No. of positive isolates**	**Percentage (%)**
*Staphylococcus*	*nuc*	270	379	285	75
	*seb*	643	285	157	55
	*hla*	535	285	88	31
*E. coli*	*E coli*	232	235	202	86
	*stx1*	180	202	177	87.21
	*stx2*	255	202	77	38.11
	*hly*	534	202	119	58.91
*Strep. agalactiae*	*S agl*	405	115	96	83.33

**Figure 4:**
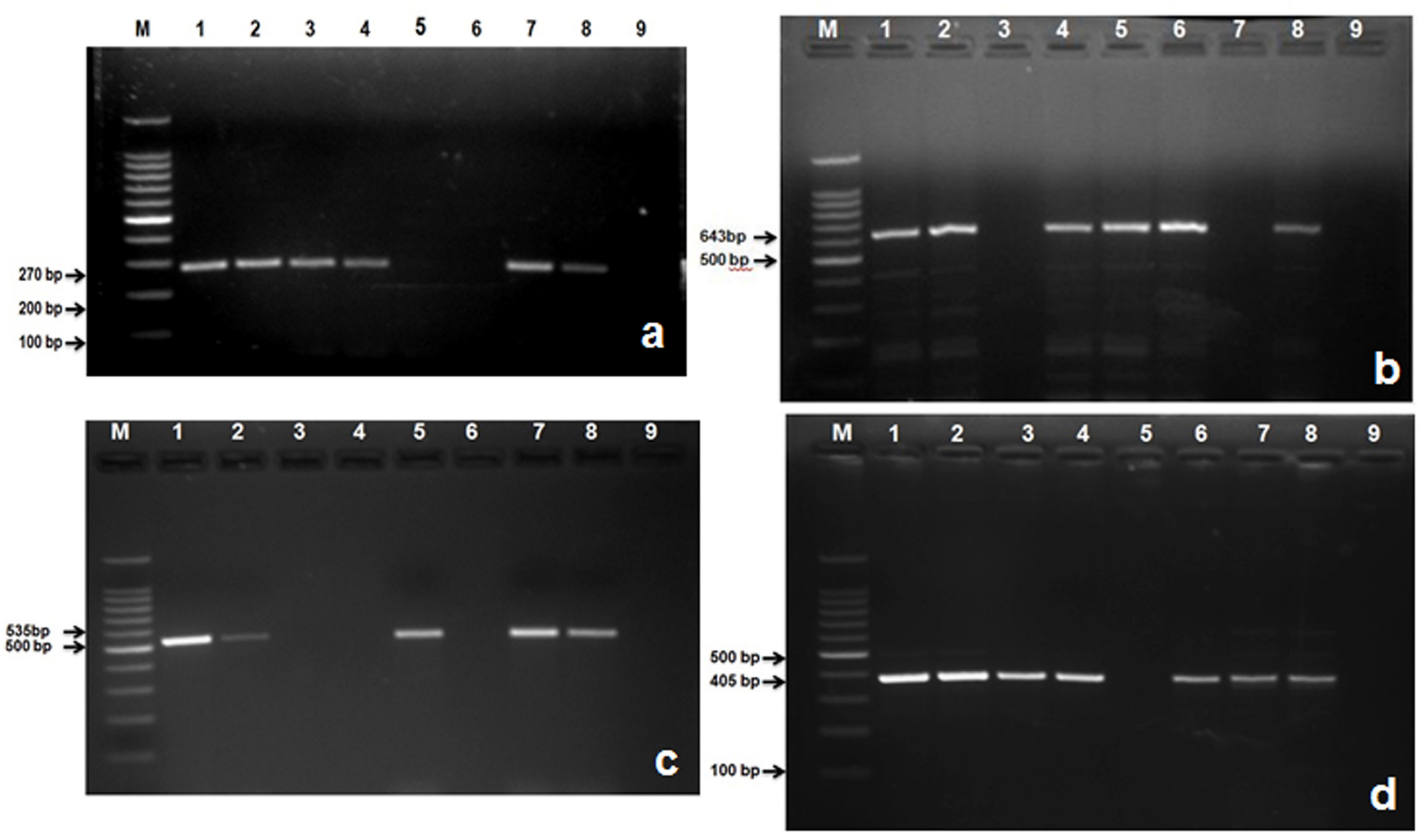
Amplified PCR products of *S. aureus* and *S. agalactiae* genes isolated from mastitis milk samples. **(a)** PCR product of *nuc* gene at 270 bp. Lane M: 100 bp ladder; Lane 1: positive control; Lanes 2, 3, 4, 7, and 8 are *nuc* gene positive; Lane 5, 6: are *nuc* gene negative; Lane 9: negative control; **(b)** PCR product of *seb* gene at 643 bp. M: 100 bp ladder; Lane 1: positive control; Lanes 2, 4, 5, 6, and 8 are *seb* gene positive; Lane 3, and 7: are *seb* gene negative; Lane 9: negative control; **(c)** PCR product of *hla* gene at 535 bp. M: 100 bp ladder; Lane 1: positive control; Lanes 2, 5, 7, and 8 are *hla* gene positive; Lane 3, 4, and 6: are *hla* gene negative; Lane 9: negative control; **(d)** Lane M: 100 bp ladder; Lane 1: positive control; Lanes 2, 3, 4, 6, 7, and 8: are *S agl* gene positive; Lane 5: is *S agl* gene negative; Lane 9: negative control.

In this study, 285 (75%) isolates out of a total of 379 gave positive results for *nuc* gene of *S. aureus* (Table 6; Figure 4a). Then the confirmed strains of *S. aureus* by PCR were subjected to their toxins gene analysis such as *seb* gene (643 bp) (Enterotoxin B) (Figure 4b) and *hla* gene (535 bp) (alpha—toxin) (Figure 4c), and it was found that 157 (55%) organisms out of 285 harbored *seb* gene whereas 88 (31%) organisms out of 285 harbored *hla* gene.

**Figure 5:**
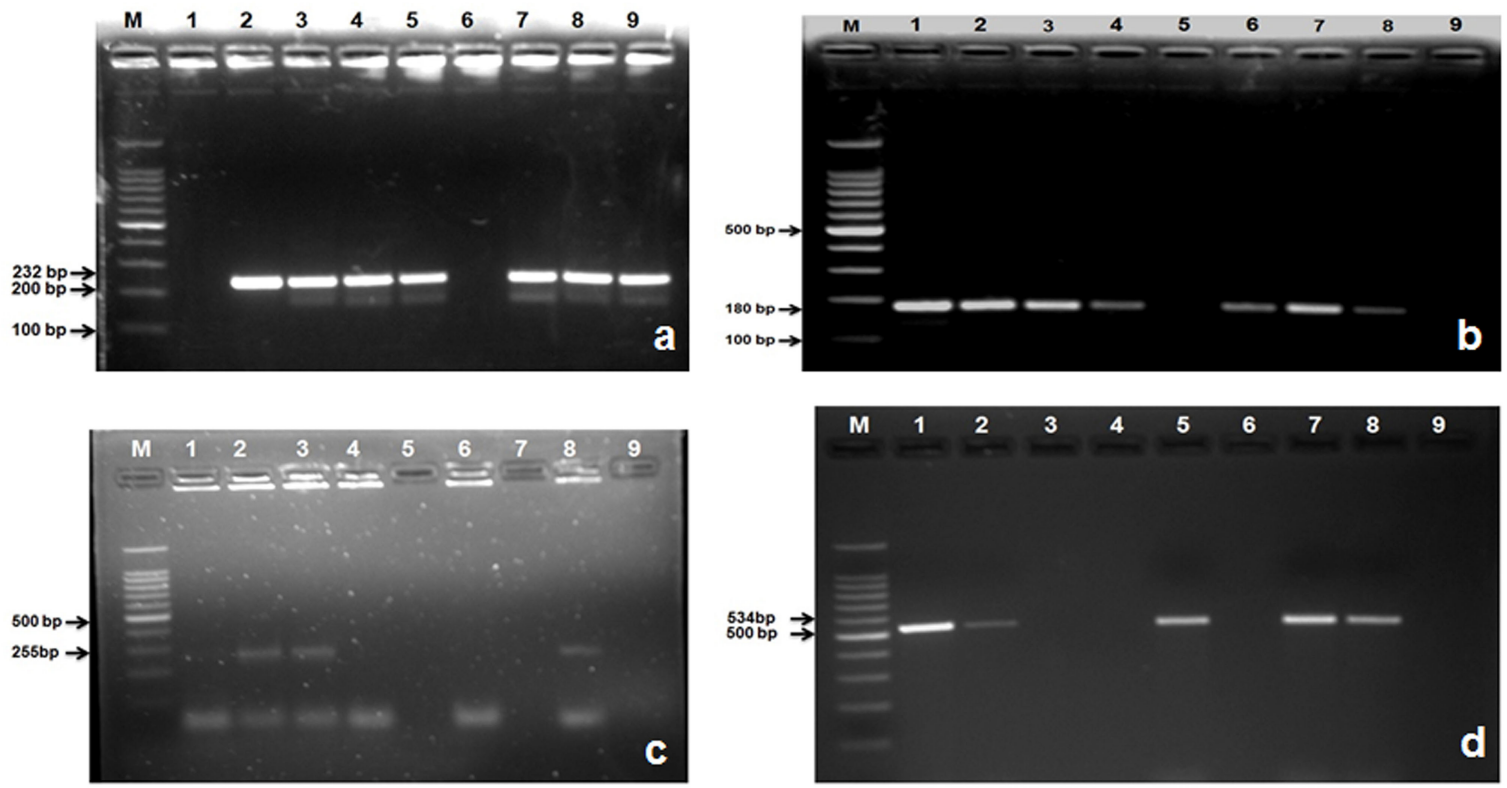
Amplified PCR products of *E. coli* gene isolated from mastitis milk samples. **(a)** PCR product of *E. coli* gene at 232 bp. Lane M: 100 bp ladder; Lane 1: negative control; Lanes 2: positive control; Lane 3, 4, 5, 7, 8, and 9: are *E. coli* gene positive; Lane 6: is *E. coli* gene negative; **(b)** PCR product of *stx1*gene at 180 bp. M: 100 bp ladder; Lane 1: positive control; Lanes 2, 3, 4, 6, 7, and 8: are *stx1* gene positive; Lane 5: is *stx1*gene negative; Lane 9: negative control; **(c)** PCR product of *stx*2 gene at 255 bp. M: 100 bp ladder; Lane 8: positive control; Lanes 2, 3: are *stx*2 gene positive; Lane 1, 4, 6, 7, and 5: are *stx*2 gene negative; Lane 9: negative control; **(d)** PCR product of *hly* gene at 180 bp. M: 100 bp ladder; Lane 1: positive control; Lanes 2, 5, 7, 8: are *hly* gene positive; Lane 3, 4, 6: are *hly* gene negative; Lane 9: negative control.

A total of 235 *E. coli* isolates from CMT positive bovine milk samples were subjected for further confirmation by their specific *E. coli* gene (232 bp); out of them, 202 (86%) showed a positive results (Table 6; Figure 5a).

After confirmation by PCR, all 202 isolates of *E. coli* were subjected for detection of Shiga toxin-producing *E. coli* (STEC) by specific gene *stx*1 (180 bp), *stx*2 (255 bp), and *hly* (534 bp) using PCR, and we found that 177 (87.21%) *E. coli* isolates were positive for *stx*1 gene (Figure 5b), 77 (38.11%) harbored *stx*2 gene (Figure 5c), whereas 119 (58.91%) were harboring *hly* gene (Table 6; Figure 5d).

A total of 115 isolates of *Strep. agalactiae* isolated from CMT positive bovine milk samples were used for further confirmation by PCR using a specific *S agl* gene (405 bp). Results showed that 96 (83.33%) isolates were positive for the targeted *S agl* gene (Table 6; Figure 4d).

## Discussion

Various pathogens are involved in the mastitis origin, ranging from bacteria to fungus. Pathogenic bacteria of bovine udder originate either as environmental pathogens or as contagious microbes. In this study, we observed that out of 1,100 isolates, 36.09% were *S. aureus* followed by *E. coli* (21.36%) and coagulase negative *Staphylococci* (13.54%). Amongst the least frequently isolated organisms were *Proteus, Corynebacterium*, and *Bacillus* spp. This study was in agreement with the findings of Bhat et al. (15), which reported *Staphylococcus aureus* as the main responsible bacteria for the clinical mastitis in bovines of Jammu, UT of Jammu & Kashmir. Majority of bacterial species isolated in our study were in accordance to the findings reported in other research studies that were conducted in Korea, Egypt, Poland, Ethiopia, and Brazil (16, 17).

However, in contrast to our study Kumar et al. (18) found that the major (50.00%) organism involved in SCM cases in cows was *Streptococcus dysagalactiae* followed by *S. aureus* and others. Sharma et al. (19) reported that both the clinical and sub-clinical mastitis were predominantly of contagious origin in their review on mastitis, emphasizing studies conducted in Asian countries. The predominance of *Staphylococcus* spp. is because of their ubiquitous nature and colonization of skin and udder. Besides a developing resistant nature, transmission at the time of milking can also be stated as possible.

### Molecular Characterization of Bacterial Isolates

On cultural examination, organisms that primed the list were *S. aureus, E. coli*, and *Strep. agalactiae*, which were further subjected to characterization and confirmation by PCR. Out of total *Staphylococcus* spp. isolates, only 75% were positive for *nuc* (nuclease) gene, which is an important virulence factor and a unique marker for *S. aureus* (20, 21). The confirmed strains were further subjected to the toxin gene analysis using PCR; *seb* gene (enterotoxin B) and *hla* (hemolysin-A). Out of all the positive samples for *nuc* gene, 55.00% and 31.00%, showed a positive results for *seb* and *hla* gene, respectively. A recent study conducted in one of the Asian countries i.e., Bangladesh found that 69.7% of isolated strains (from pure colonies) were positive for at least one enterotoxin gene, with one amongst them even presenting a combination of six genes (pvl, sea, seb, sec, sed and see) (22). On the contrary, Bastos (23) reported that the most common enterotoxin of *S. aureus* involved in the food poisoning outbreaks is the *sea*. The differences in the isolation of enterotoxins may be due to the differences in the ecological reservoirs of *S. aureus* in different countries and regions of the world. Alpha-hemolysin (*hla*) toxin is the most emphasized and characterized virulence factor of *S. aureus* (24). It contributes to the pathogenesis of *S. aureus* infection, including that of cell signaling pathways that govern cell proliferation, cytokine secretion, inflammatory responses, and cell–cell interactions (25, 26).

The confirmed *E. coli* were further subjected to the detection of Shiga toxin-producing *Escherichia coli* (STEC) and hemolysin gene by using specific genes *stx1, stx*2, and *hly* by PCR; and found 87.21%, 38.11, and 58.91%, respectively, positive for these toxic genes. These results agreed with Momtaz et al. (27), who found that the *stx*1 gene had the highest prevalence in *E. coli* isolated from bovine mastitic milk. On the contrary, others had mentioned that *E. coli* strains isolated from cows with clinical mastitis were negative for both *stx*1 and *stx*2 genes (28). Most *E. coli* serotypes isolated from mastitic cows and buffaloes produced verotoxin, hypothesized to efficiently inhibit protein synthesis in the mammalian cell-free system (29) and the presence of hemolysin gene present in mastitic milk is responsible for hemolytic activity (30). Shiga toxins are major virulence factors encoded by Shiga toxins (*Stx*1) and (*Stx*2) genes; these genes are located in the genome of temperate bacteriophages. All these studies highlighted that the distribution pattern of Shiga toxins among *E. coli* varies from region to region. Several studies showed that STEC isolates are an important group causing mastitis (31).

### Milk Somatic Cell Count and Composition

Clinical mastitis causes visible changes in the milk, like clots, flakes, blood, and severely affects the milk quality. For quality control, the increase of somatic cell count in milk is considered an optimum marker for detecting mastitis (32). The present study revealed that with the increasing severity of mastitis, confirmed by CMT grading from “0” to “3,” the milk SCC also increased from 96.00 ± 3.60 (× 10^3^/ml) to 1738.70 ± 79.40 (x10^3^/ml). An elevated level of SCC in milk has negatively influenced the quality of raw milk (3). SCC is a valuable predictor of intramammary infection and helps assess the aspects of quality, hygiene, and mastitis control. During intramammary infection, the major increase in SCC is due to the influx of over 90% neutrophils into the milk to fight infection (33).

A significant decrease in the percentages of fat, lactose, protein, ash, and SNF in milk was found in drawing a correlation between SCC and the milk composition. A recent study has also reported that increased SCC negatively affects milk quality and composition (34).

Mastitis has a huge impact on total milk output and modification of its composition and technological usability. Mammary epithelial cells can be damaged by bacteria, resulting in a reduced ability to synthesize milk components (1). Reduction in milk lactose concentration has been seen (35), mainly due to the destruction of the normal lactose barrier, causing leakage of lactose into the extracellular fluid and blood (36). Ogola et al. (37) investigated and found three mechanisms involved in milk composition change due to mastitis: decreased synthesis, increased permeability of the milk barrier, and increased proteolytic activities in milk (38). The low lactose concentrations depend on the severity of damage to the tight junctions (39) caused by the different bacterial strains involved. In this study, we also found that milk fat significantly decreased with the severity of mastitis. Decrease fat content during mastitis can be awarded to the mammary gland's reduced synthetic and secretory capacity (40).

Like our findings, other studies have also reported a decrease in milk lactose concentration (39, 41). The reduced lactose in affected quarters probably is due to impaired synthetic activity or to the damaging effects of pathogens on the mammary parenchyma, and preferably the reason for depressed concentrations is the proliferation of paracellular pathways that lead to leakage of lactose out of milk (42). Therefore, the severity of damage to the tight junctions leads to low lactose concentrations (39) caused by the different bacterial strains involved.

## Conclusions

Based on the cultural examination, *S. aureus* was found to be the most prevalent etiological microorganism present in mastitis-affected milk, followed by *E. coli*, coagulase negative *Staphylococci*, and other environmental pathogens. The major bacteria such as *S. aureus, E. coli*, and *Strep. agalactiae* showed various toxic genes responsible for the pathogenesis of mastitis and disease conditions in consumers of milk and milk products. SCC was found highest in the grade 3 score of CMT. In correlation with milk components, it was found that SCC had a significant negative correlation with fat, lactose, ash, SNF, whereas the correlation with protein was non-significant and negative. It was concluded that mastitis significantly reduced the quality of milk by decreasing fat, protein, and lactose and contained pathogenic microorganisms.

## Data Availability Statement

The original contributions presented in the study are included in the article/supplementary material, further inquiries can be directed to the corresponding authors.

## Author Contributions

NS and SL conceived and designed the experiments and analyzed the data. ZH and SK performed the experiment and writing the manuscript. All authors contributed to the article and approved the submitted version.

## Funding

This work was supported by the Department of Biotechnology, Government of India, New Delhi, India (Project No.: BT/PR21547/NNT/28/1232/2017) and National Research Foundation Project Grant No.: 2017R1A2B2012125, Republic of Korea.

## Conflict of Interest

The authors declare that the research was conducted in the absence of any commercial or financial relationships that could be construed as a potential conflict of interest.

## Publisher's Note

All claims expressed in this article are solely those of the authors and do not necessarily represent those of their affiliated organizations, or those of the publisher, the editors and the reviewers. Any product that may be evaluated in this article, or claim that may be made by its manufacturer, is not guaranteed or endorsed by the publisher.
